# Bringing bioinformatics to schools with the 4273pi project

**DOI:** 10.1371/journal.pcbi.1009705

**Published:** 2022-01-20

**Authors:** Stevie A. Bain, Heleen Plaisier, Felicity Anderson, Nicola Cook, Kathryn Crouch, Thomas R. Meagher, Michael G. Ritchie, Edward W. J. Wallace, Daniel Barker

**Affiliations:** 1 Institute of Evolutionary Biology, School of Biological Sciences, University of Edinburgh, Edinburgh, United Kingdom; 2 Institute for Cell Biology and SynthSys, School of Biological Sciences, University of Edinburgh, Edinburgh, United Kingdom; 3 Centre for Biological Diversity, School of Biology, University of St Andrews, St Andrews, United Kingdom; 4 Wellcome Centre for Integrative Parasitology, University of Glasgow, Glasgow, United Kingdom; McGill University, CANADA

## Abstract

Over the last few decades, the nature of life sciences research has changed enormously, generating a need for a workforce with a variety of computational skills such as those required to store, manage, and analyse the large biological datasets produced by next-generation sequencing. Those with such expertise are increasingly in demand for employment in both research and industry. Despite this, bioinformatics education has failed to keep pace with advances in research. At secondary school level, computing is often taught in isolation from other sciences, and its importance in biological research is not fully realised, leaving pupils unprepared for the computational component of Higher Education and, subsequently, research in the life sciences. The 4273pi Bioinformatics at School project (https://4273pi.org) aims to address this issue by designing and delivering curriculum-linked, hands-on bioinformatics workshops for secondary school biology pupils, with an emphasis on equitable access. So far, we have reached over 180 schools across Scotland through visits or teacher events, and our open education resources are used internationally. Here, we describe our project, our aims and motivations, and the practical lessons we have learned from implementing a successful bioinformatics education project over the last 5 years.

## Introduction

Rapid advances in technology over recent decades have changed the practice of research across all areas of the life sciences. In particular, with the advent of high-throughput, next-generation sequencing technologies, biological research has become more data driven and digital [1]. Bioinformatics—a discipline combining aspects of biology, computing, mathematics, and statistics—is now an essential element of modern biology. Increasingly, over the last few decades, researchers have been required to understand and utilise computational tools to store, manage, and analyse biological data [2,3]. Therefore, biology graduates with bioinformatics training and experience have increased employment opportunities [4] and are better prepared for postgraduate research in life sciences fields [5].

Despite this, bioinformatics education has failed to keep pace with advances in life sciences research [5–7]. In general, much bioinformatics instruction takes place at postgraduate level, e.g., MSc courses and doctoral training programmes [8], meaning that many life sciences students can earn their degrees with little or no bioinformatics training [5,9]. In the last few years, there has been a push to incorporate bioinformatics into undergraduate curricula [10–13]; however, a recent study found that one of the key barriers to this integration is a lack of student preparation [9].

Introducing bioinformatics at secondary school level may resolve this issue. It has been suggested that the earlier computational skills are embedded, the more likely students are to retain this knowledge and their confidence in the subject will increase [14]. Indeed, a number of school-level bioinformatics education programmes and initiatives have been designed with this goal in mind (e.g., [15–17]). In Scotland, unlike in the rest of the UK, bioinformatics is included in the biology curricula for both Higher level Biology and Human Biology [18,19]. These are senior-level qualifications open to pupils usually aged 15 to 18 years old and are required for entry to Higher Education. However, the mention of bioinformatics in the curricula and in the course-recommended textbooks [20,21] is brief. Therefore, while pupils are expected to know the term “bioinformatics” and its relationship to DNA sequencing, there is no formal requirement for practical experience and contextualisation.

To address the disparity between future bioinformatics workforce needs and the level of bioinformatics training at secondary school level, we designed two curricula-linked workshops that introduce pupils (and teachers) to the field of bioinformatics and also reinforce other key topics in the biology curricula such as mutation. In this paper, we describe our educational resources, our approach in targeting our audience, and summarise our successes and limitations over the duration of the project. We also discuss the practical lessons learned from running a successful bioinformatics education project for schools in Scotland.

## 4273pi bioinformatics education project

### Our workshops

**Fig 1:**
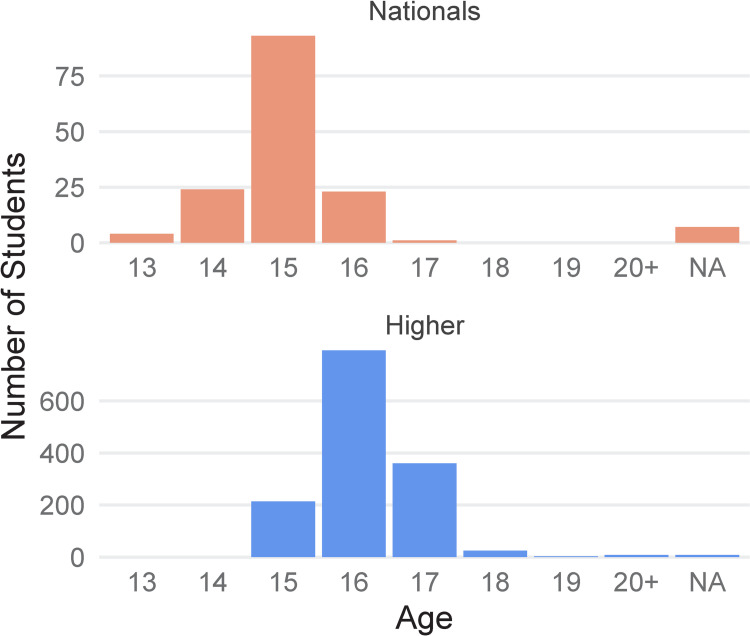
Age range of individuals taking part in the bioinformatics workshops. Nationals graph (top, orange) shows the age range of 152 pupils that have taken part in our SQA National 4/5 level workshop, Bioinformatics: Food Detective. Higher graph (bottom, blue) shows the age range of 1,412 pupils that have taken part in our SQA Higher level workshop, Bioinformatics: The Power of Computers in Biology. This is not the total number of individuals that have participated in the Higher workshop but a subset that filled out evaluation forms. These data represent workshops led by our project staff or volunteers.

A key reason for the success of our project is our strong link to the Scottish Qualifications Authority (SQA) Biology curricula. As well as bringing practical bioinformatics to the classroom, we incorporate other key topics from the curricula to reinforce learning, e.g., mutation and nutrition. We have designed 2 different workshops to fit in with the Scottish biology curricula. Our workshop for pupils in lower secondary school stages—the National level workshop—was designed with the SQA National 4 and National 5 level curricula in mind [22]. These qualifications are open to pupils usually aged 13 to 16 years old, and they are often required for progression to the SQA Higher level qualifications. Our workshop for senior secondary school pupils and students in Further Education (FE)—the Higher level workshop—was designed with the SQA Higher Biology and Human Biology curricula in mind. Fig 1 shows the ages of individuals reached through each of these workshops.

Both workshops use freely available online research resources: the National Center for Biotechnology Information (NCBI) database and its BLAST tool [23,24] and are built on case studies involving DNA sequences. The Higher level workshop additionally introduces the Linux command-line using Raspberry Pi computers. These are small computers designed initially with the aim of promoting the study of computer science in school. They are inexpensive and easily transportable, making them ideal for school visits. We preinstall each Raspberry Pi with the 4273pi variant of the Raspbian operating system (named after a former undergraduate module at the University of St Andrews) [12], and all required software and files. Internet or school network access is not required on these machines during the workshop, as such, their use keeps the focus on the advantages of command-line computing and away from potential IT problems. The Raspberry Pi is important for our workshops but is also ideally suited to bringing a range of computational biology activities to school [25]. Furthermore, these computers are also used for research, meaning that pupils can gain hands-on experience of an authentic computational research environment in their own classrooms.

### Bioinformatics: The power of computers in biology

We designed our Higher level workshop based on materials originally developed by DB for first-year undergraduate biology students at the University of St Andrews. This workshop has 2 major aims. The first is educational: We want to help young people understand the role of bioinformatics and the interdisciplinary links between computing, mathematics, and biology by exploring these themes in the context of the SQA Biology and Human Biology curricula, including mutations, genomics, and evolution. The second aim is public engagement: We use bioinformatics as an introduction to techniques in computational science. A full description of this workshop is available in [26]. Feedback from both teachers and pupils suggests that this workshop was beneficial and supports our hypothesis that practical bioinformatics activities can be conducted by school pupils.

### Bioinformatics: Food detective

Our more recently developed workshop is designed for pupils studying SQA National 4 and National 5 level Biology, providing an opportunity to influence the study and career choices of young people at an earlier stage of their education [22]. The workshop is based on real DNA sequences extracted from a handmade pork sausage as a case study in metabarcoding. Pupils use publicly available tools and data to discover that the sausage contains pig DNA but also traces of sheep, chicken, cattle, and human DNA. This informs pupils about a range of subjects including data reliability, contamination, and food fraud. As with our Higher workshop, feedback from both teachers and pupils suggests that this activity is useful and enjoyable. A full description of the workshop and evaluation is reported in [22].

Active learning is known to increase student performance in science, technology, engineering, and mathematics (STEM) subjects [27]. Therefore, both of our workshops aim to maximise pupil participation by asking them to undertake tasks in pairs or small groups, completing the accompanying worksheet as they go along. We also come together as a class between tasks to discuss pupils’ findings and to consider the topics covered in more detail. For example, “What happens if humans do not get enough Vitamin C?” and “Why might human DNA be found in the sausage sample?” Overall, the aim of our project is to provide pupils and teachers with hands-on experience of computing in biology and increase understanding of why it is essential in many areas of life sciences research.

While we provide lesson plans for each of our workshops based on how we run our school visits, we also encourage teachers to use our resources in whichever way best suits their needs. This is beneficial for teachers as they can fit certain activities into preexisting lessons, for example, teachers have reported that they used our Food Detective workshop alongside a DNA extraction practical. They felt that our materials complemented the existing practical lesson and reinforced curriculum learning objectives. We believe that creating resources, which can be used flexibly, either fully or in part, can increase teacher ownership of the lesson. Thus, our resources feel less “outsourced” and more embedded in the delivery of the course material.

Throughout the development of this project, we have made use of the advice provided by others involved in school-level bioinformatics education such as [28]. We have created a list of lessons learned and tips for others interested in developing their own school projects in Box 1.

**Box 1. Lessons learned and tips for others**

- Strong links to the curriculum are vital. Develop interesting, real-life case studies that pupils can relate to.
- Engage with teachers while creating and delivering workshops as they have a good understanding of what content will work for their pupils.
- Participate in action research—use experience and feedback to develop resources and make changes when needed.
- Provide training and resources for teachers. This will increase project reach and legacy.
- On school visits, work with teachers. Make sure a teacher is present to cover matters such as pupil well-being or requests to leave the classroom.
- Embed computing activities into the school biology curriculum to help address gender gaps and improve access and exposure to this traditionally male-biased subject.
- Have a diverse delivery team. For example, a mixed-gender team demonstrates the important points that computing is not “just for boys” and biology is not “just for girls.” Other dimensions of diversity are just as important.

### Action research

Action research is a solution-oriented methodological approach commonly used in the social sciences. It is a reflective and often collaborative process where researchers engage in a cycle of posing questions, gathering data, critically reflecting on findings and experiences, and implementing practical changes based on reflection [29]. We use our experiences in the classroom with pupils and teachers, and formal feedback to influence the development and delivery of our resources. We found that strong partnerships with schools and teachers are key to successfully implementing action research. Teachers have a thorough understanding of school curricula, the level at which activities should be pitched, and what activities pupils will learn from and enjoy. Therefore, incorporating teacher feedback throughout the workshop design and delivery process has been key to the success of our project.

Action research was used in the development of both of our workshops. We piloted the Food Detective workshop with junior pupils in several Scottish schools. Initially, we were concerned that the workshop may be too challenging for pupils; however, we found that pupils performed well and enjoyed the activities. They also managed to complete the workshop more quickly than we had anticipated, and in their feedback, both teachers and pupils requested more activities. We added a second task to this workshop, which focused on e-values and the reliability of results. In subsequent workshops, we found that pupils were more challenged but still engaged. The feedback showed that they enjoyed both tasks and teachers felt that the activities were pitched at the correct level for this age group. In this case, action research helped us to refine the content of our workshop based on the interests and abilities of pupils.

### Delivery of workshops and dissemination of resources

The main challenges to incorporating bioinformatics into the secondary school curriculum are infrastructure, logistics, and teacher expertise. Our project aims to address these issues through a number of actions. The Nationals workshop and two-thirds of the Higher workshop (i.e., the non-Raspberry Pi-based sections) can be carried out on any device that has access to the internet, including school desktop computers, tablets, or smartphones. Many local authorities across Scotland are now providing pupils with tablet devices for use in the classroom, which means that most of our activities can be carried out without the need to book a computer suite or library, reducing the organisational burden for teachers and schools.

We learned that to effectively reach large numbers of pupils without potential audience bias and exclusion that may occur through self-selection for extracurricular activities, whole-class visits during the school day are best. In the beginning, our workshops extended across 4 periods in the school day (approximately 3 hours 20 minutes); we later compressed this to 3 periods. However, we became aware of the disruption this caused to the school timetable and decided to adjust the duration to fit within a timetabled double period of biology (approximately 1 hour 40 minutes). We now avoid negatively impacting on other subjects and have eased the logistical burden teachers face when inviting us to their schools. We also visit all classes for any given qualification. For example, if a school has 5 Higher Biology classes, we arrange to deliver the workshop to all these classes. This approach ensures that we reach the maximum number of pupils possible.

In addition to our school workshops, our programme of free Continuing Professional Development (CPD) workshops for teachers provides an excellent opportunity to expand the reach of our project and strengthen our partnerships with schools. Teachers often appreciate the opportunity to gain a better understanding of bioinformatics and our resources with their colleagues before using them with their classes. We also contribute to initial teacher training for students enrolled in Professional Graduate Diploma in Education courses for STEM subjects. We provide teachers and student teachers with training that will enable them to deliver our workshops to their classes, which helps to increase the reach of our project. We also provide additional training on how bioinformatics can be implemented in other areas of the biology curricula, e.g., how to use the NCBI database to find the amino acid sequence of an enzyme discussed in class. Our school workshops and teacher training are delivered by a core team of staff and trained volunteers from several universities across the UK.

When coordinating events for teachers, collaborating with educational institutions (in our case, Scottish Schools Education Research Centre (SSERC) or the Science Skills Academy of Highlands and Islands Enterprise (SSA)) and local authorities is beneficial. These institutions can provide support such as venues, access to school mailing lists, and details of in-service and teacher training days.

### The importance of online and open education resources

Open education resources (OERs) have a key role in ensuring inclusive and equitable quality education opportunities for all (United Nations Sustainable Development Goal 4) [30]. The Coronavirus Disease 2019 (COVID-19) pandemic has further highlighted their importance at both a local and global level. Our workshops are free to attend, and our materials are available as OERs, removing financial burdens from teachers and schools. To further extend our reach, we have also published Practical Guides for each of our workshops with GOBLET, the Global Organisation for Bioinformatics Learning, Education, and Training [26,31]. GOBLET provides high-quality, open-source resources to support bioinformatics learning, education, and training across the world [2]. There are resources designed to cover all levels of bioinformatics training from schools to research laboratories, and thus this partnership allows us to increase our audience reach.

In response to the COVID-19 pandemic, we developed several additional online resources that made it possible for us to continue to support and encourage teachers to incorporate bioinformatics into biology lessons. In addition to their usefulness throughout the pandemic, these resources have demonstrated the potential to increase the reach and scalability of our project in the future. We created a teacher-oriented video series designed to support teacher CPD (https://4273pi.org/videos). These short videos (5 to 10 minutes) run through the key aspects of our workshops and also provide information about using the NCBI database in other areas of biology curricula, e.g., using the NCBI 3D protein viewer to examine the structure of enzymes. All videos have subtitles and accompanying transcripts to increase accessibility.

Feedback from pupils, teachers, and STEM education professionals shows that our workshops and OERs are useful and effective learning tools. As a testament to this, our workshops and videos have been added to a list of online OERs compiled by Education Scotland to support teachers with online learning.

We also adapted our in-person CPD event for teachers to an online delivery model. Discussions with teachers and colleagues from Education Scotland indicated that twilight sessions (between 4.30 to 6.30 PM) would maximise teacher participation without disrupting the school day. In addition, we offer CPD during teacher in-service days. In advance of each CPD event, we send teachers a resource kit containing a Raspberry Pi computer complete with SD card image customised for bioinformatics, plus a case, power supply, and monitor cables; an introductory bioinformatics textbook [32]; and a hard copy of our handouts (these are also available electronically). As with our in-person CPD, these events and resource kits are free, and our aim is to provide teachers with the equipment, knowledge, and confidence required to integrate bioinformatics into their classroom teaching. At some CPD events, we also host guest speakers who present case studies of their own computational biology research. Even when we resume in-person CPD, we will also continue to provide a programme of online CPD events, as these allow us to reach a larger audience more easily, particularly in remote areas such as the Highlands and Islands region of Scotland. Our online CPD events also eliminate any travel costs associated with attending, which is important as many schools have limited budgets and costs can be substantial for teachers, especially those in remote areas.

### Project reach and prioritising socially disadvantaged areas

Every education project should have a clearly defined target audience, one that will benefit from the resources produced and that is logistically feasible to reach. Our primary focus is Scotland, for several reasons. The first reason is that bioinformatics is included in the Scottish Biology curriculum, meaning that we can create resources that link directly to course content in schools. Secondly, Scotland provides a tractable target audience that our project can easily reach without the need for satellite groups. By focusing our efforts on a specific geographical area, we can achieve fuller coverage and build relationships with schools. Additionally, there is only one Scottish exam board, the SQA, and all Scottish schools are supported by the same Government agencies, e.g., Education Scotland.

**Fig 2:**
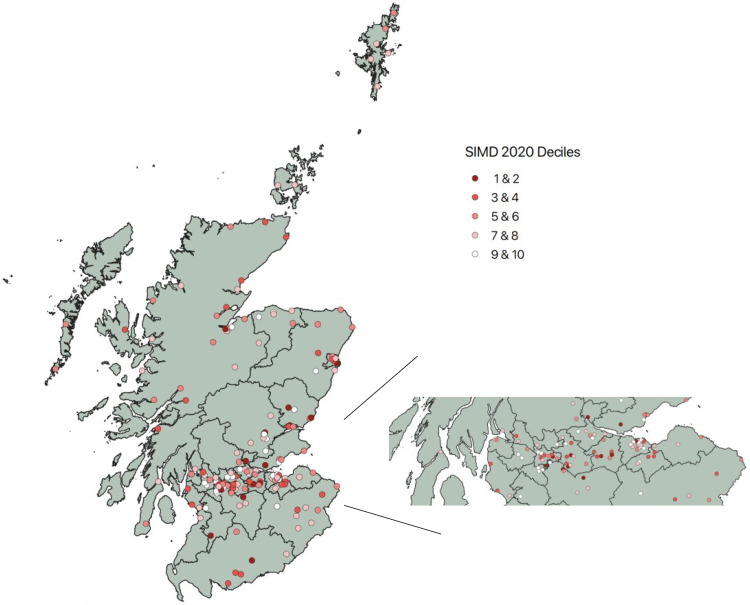
A map of Scotland showing the schools our project has reached since 2016. Each school reached is represented by a coloured circle on the map. Some schools have been reached more than once. Circles are coloured according to Scottish Index of Multiple Deprivation 2020 (SIMD) decile (1—most deprived to 10—least deprived) [36]. Dark red circles represent schools in the most deprived areas of Scotland according to SIMD, and the white circles represent schools in the least deprived areas. A gradient colour scheme represents those areas in between. The zoomed-in section shows the densely populated central belt region with more clarity. (Map shape file downloaded from https://www.nrscotland.gov.uk/statistics-and-data/geography/our-products/census-datasets/2011-census/2011-boundaries. Contains NRS data Copyright Crown copyright and database right 2021. Contains Ordnance Survey data Copyright Crown copyright and database right 2021.

We have directly reached over 180 Scottish secondary schools since 2016 (Fig 2), including almost 50% of state secondary schools. We advertise our project across Scotland via a biology teacher e-mail list and ask any interested teachers to get in touch with us. We also have strong working relationships with organisations such as Education Scotland, SSERC, and SSA that have supported us by facilitating our in-person CPD events (SSERC and SSA) and contributing to the coordination of our online CPD events. Although our primary focus is Scotland, our OERs are used internationally (e.g., elsewhere in the UK, Mexico, Argentina, India, the USA, Finland, Brazil, China, the Netherlands, and Saudi Arabia).

Science and technology can be inspiring to all; however, we know that the playing field is not level [33]. One set of systemic biases prominent in STEM sectors is discrimination against socioeconomically disadvantaged groups [34]. These inequalities are also seen at the school level where socioeconomically disadvantaged pupils, despite being interested in science, have fewer opportunities to engage with science both inside and outside of school [35].

We often have a waiting list for our visits and, initially, we used to target specific geographical areas each year to maximise our coverage of Scotland. However, we know that there are areas in the country where pupils do not have the same opportunities to explore and engage with science and technology as others. Therefore, we now use the Scottish Index of Multiple Deprivation (SIMD) [36] to prioritise our school visits. The SIMD is a relative measure of deprivation across Scotland. In this context, “deprived” is not synonymous with poor, and the SIMD is used to describe areas, not people. However, those who live in the “most deprived” areas are more likely have limited access to Higher Education Institutes and public engagement opportunities. The index looks at 7 domains relevant to resources and opportunities (income, employment, education, health, access to services, crime, and housing) within a certain geographical area, called a datazone. The SIMD is used by the Scottish Government and funding bodies to effectively target policies and funding to areas where deprivation is high. We target low SIMD areas (i.e., those most deprived on the SIMD scale), which often include postindustrial towns and cities. Initially, our project used the SIMD of the school itself to target visits, by using the school’s postcode. However, we now recognise that this is not a perfect proxy for targeting pupils that come from low SIMD areas. For example, some pupils in a school will almost certainly live in areas that have a different SIMD score than the score of their school, particularly if the school is in a city centre. To address this, we have recently focused our efforts into targeting schools that have a high proportion of pupils who actually live in low SIMD areas. Publicly available data from the Scottish Government (available from: https://www2.gov.scot/Topics/Statistics/Browse/School-Education/Datasets/contactdetails) showed 42 secondary schools in Scotland where >50% of pupils attending lived in SIMD deciles 1 and 2 (most deprived areas in Scotland). From these data, we have identified areas and schools that we will target for future visits. By targeting low SIMD schools for our teacher CPD and school visits, we can promote our OERs and support schools to access and use them. Equitable access to OERs is essential to ensure that these resources will overcome existing inequalities rather than entrench them [37]. It is important to note that our workshops are not designed specifically for or delivered only to low SIMD areas, but we do believe it is important to prioritise schools where public engagement and university contact is less likely to happen.

As well as the SIMD, we also take into consideration the location of schools. Approximately 17% of Scotland’s population live in areas defined as rural, and approximately 20% of Scotland’s secondary schools are rural [34,35]. Although not necessarily classified as “most deprived” datazones in overall SIMD analyses, rural areas often have reduced access to public engagement and education events hosted by Higher Education institutes compared to nonrural areas [36,37]. The commission on the Delivery of Rural Education 2013 [38] recommended to the Scottish Government that Further and Higher Education institutions, local authorities, and schools should work together to provide the widest possible range of opportunities to young people and adults in rural areas. Another recommendation was to provide access to CPD to teachers in rural areas, reducing isolation and sustaining skills and development. At an early stage of the project, the strong demand for CPD in locations outside the Scottish central belt became apparent to us through direct contact with teachers. Therefore, each year we run events that specifically target schools and teachers in the Highlands and Islands regions. Our online CPD events allow us to reach these teachers and schools more effectively.

### Addressing the gender bias in computing

In the UK and some other countries, computer science education is associated with a startling gender inequality (Table 1) [38]. Ironically, computer programmers were initially predominantly female, partly due to the accelerated development of computer technology during World War II [39,40]. Later, computing became male dominated, due to misguided efforts of government and industry in the 1960s to 1980s to bring the topic to a managerial (i.e., male) level [41]. In the UK, only 18% of those studying a computing science at university level are female [38]. Importantly, it should be noted that this bias is not common to all countries, for example, in India, women represent 45% of total computer science enrolment in universities [42]. Early introduction of computing sciences at primary and secondary school level is suggested to have a significant impact on gender representation at university level [42]. In Malaysia, gender ratios in computer science education are not merely balanced; sometimes they are dominated by women [43]. Again, early computer science education is noted as a factor influencing these patterns [43]. This highlights the fact that more can and needs to be done to remedy the strong gender bias that exists in Scotland, the UK, and in many other countries. There is strong evidence to suggest that the earlier computer science education is incorporated into learning, the better this bias is addressed [14]. We also believe that school engagement and educational programmes must look beyond the obvious audience of pupils already engaged with computing education (male biased; see Table 1) and even beyond the audience of young people with any self-declared interest in computing.

**Table 1:** Percentage of female pupils at different levels of Biology and Computing across Scotland.

Subject	SQA level	SCQF level	Percentage of pupils that are female
Biology	National 4	4	63%
	National 5	5	67%
	Higher	6	66%
Computing	National 4	4	16%
	National 5	5	15%
	Higher	6	16%

These figures are taken from the SQA Statistical Report 2018 [44]. The figures clearly show that Biology is a female-biased subject and that Computing is the opposite with a striking male bias. Therefore, we believe that by targeting Biology classes, we are bringing computation to an audience that would not necessarily receive this education. SCQF levels indicate the difficulty of particular subjects in the Scottish education system. These are comparable to the RQF and CQFW in England and Wales, and the EQF. SCQF levels 4, 5, and 6 are comparable to RQF and CQFW levels 1, 2, and 3, respectively, and EQF levels 2, 3, and 4, respectively (more detail available at https://www.sqa.org.uk/sqa/64561.html).

CQFW, Credit and Qualifications Framework; EQF, European Qualifications Framework; RQF, Regulated Qualifications Framework; SCQF, Scottish Credit and Qualifications Framework; SQA, Scottish Qualifications Authority.

Our project specifically targets biology classes; approximately 66% of pupils enrolled in SQA Higher Biology are female [44]. Furthermore, 63% and 67% of pupils enrolled in SQA National 4 and 5 level Biology, respectively, are female [44]. In our own data, we see that 69% of our pupil audience identifies as female. This is a reversal of the strong gender bias associated with computing classes. SQA National 4 and 5 Computing classes are only 16% and 15% female, respectively. SQA Higher Computing classes are 16% female. Our project has the potential to inform and influence the future career goals of these pupils, thus helping to address the extreme gender gap at later career stages in computer sciences. Furthermore, feedback from pupils shows that there are no significant differences in enjoyment and usefulness of our workshops between the genders [22]. At educational levels where pupils have a choice of subjects, targeting biology classes—rather than computing classes—will have advantages for reducing gender bias in bioinformatics.

### Project impact and plans

Looking to the future, we aim to increase our reach and to focus particularly on schools with a high percentage of pupils from low SIMD areas, as we realise that these often miss out on interactions with Higher Education Institutes. We also hope the project can play a role in increasing the participation of students from underrepresented backgrounds (e.g., social class, first-generation students, and ethnic minorities, to name a few) in Higher Education. In particular, we aim to increase female pupil exposure to computational biology, highlighting the field as a potential career avenue.

In addition, we plan to develop a workshop for pupils studying computing and target this workshop at early secondary school pupils, when computing is still compulsory. Our aim is to highlight that computation is far from an insular subject. It has many diverse applications, and the storage and analysis of big data in biology provide an excellent example of this. As computing is increasingly central to many areas of life, bioinformatics is increasingly central to life sciences. We are proud to be part of the effort to bring bioinformatics to young people.
